# Isolation and Characterization of Novel Biological Control Agent *Clostridium beijerinckii* against *Meloidogyne incognita*

**DOI:** 10.3390/biology11121724

**Published:** 2022-11-28

**Authors:** Xinglong Lian, Shuang Liu, Lanyuwen Jiang, Xinyu Bai, Yuanyuan Wang

**Affiliations:** 1 College of Bioscience and Biotechnology, Shenyang Agricultural University, Shenyang 110866, China; 2 Nematology Institute of Northern China, Shenyang Agricultural University, Shenyang 110866, China

**Keywords:** *Clostridium beijerinckii*, *Meloidogyne incognita*, rhizosphere, biocontrol, biomass

## Abstract

**Simple Summary:**

*Meloidogyne incognita* is one of the most widely distributed and researched species of *Meloidogyne*, which has caused enormous economic losses for Chinese agriculture. Biological control is a nonhazardous method of pest and pathogen management. Nematophagous bacteria, which are ubiquitous and have extensive host ranges, are an efficient natural adversary of nematodes. Thus, to find novel, promising biological control agents for RKNs, we conducted trials to isolate and screen effective bacterial endophytes against *M. incognita,* and then we selected an effective strain using morphological and molecular approaches. Furthermore, a greenhouse experiment was conducted to assess their potential as biocontrol agents against *M. incognita* and to investigate the effect of several strains on increasing seed germination and tomato plant growth. Strain Sneb518 (*Clostridium beijerinckii*) was identified as having solid biocontrol activity against *M. incognita*. Sneb518 demonstrated significant inhibition against *M. incognita*, with J2 mortality reaching 90.73% at 12 h and with eggs hatching at a rate of 6.00% at 24 h. Additionally, Sneb518 was excellent for enhancing seed germination. The effectiveness and stability of *M. incognita* management by Sneb518 *C. beijerinckii* were further evaluated in a greenhouse. This research will offer insightful information on the application of Sneb518 as a biocontrol agent for RKN management.

**Abstract:**

One of the most severe soil-borne pathogens in the world is the root-knot nematode (*Meloidogyne incognita*). Biological control is gaining more importance as environmental awareness increases. Thus, keeping this in mind, a total of 712 bacterial strains were isolated from 117 rhizosphere soil samples and investigated for potential biological control activity against *M. incognita*. Strain Sneb518 (*Clostridium beijerinckii*) was identified as having solid biocontrol activity against *M. incognita*. Sneb518 demonstrated significant inhibition against *M. incognita*, with J2 mortality reaching 90.73% at 12 h and with eggs hatching at a rate of 6.00% at 24 h, compared to a hatchability level of 29.07% for the control. Additionally, Sneb518 was excellent for enhancing seed germination. The seeds coated with a fermentation broth containing Sneb518 efficiently boosted the germination rate to 88.49%. The effectiveness and stability of *C. beijerinckii* Sneb518 against *M. incognita* were then further evaluated in a greenhouse. According to the pot experiment data, Sneb518 considerably (*p* < 0.05) reduced the number of root galls and egg masses on roots and also significantly (*p* < 0.05) increased tomato plant growth. *C. beijerinckii* Sneb518-treated tomato seedlings exhibited 50.26% biocontrol effectiveness compared to the control group. Our results demonstrate that *C. beijerinckii* Sneb518 can be a potential biological control agent against root-knot nematode disease and a biomass enhancer. This research will give new options for the sustainable control of root-knot nematode disease in tomatoes and other host plants.

## 1. Introduction

Plant-parasitic nematodes (PPNs) pose a considerable risk to a wide range of crops, causing global yield losses [1,2]. They may easily damage crops by feeding on them and forming associations with other microorganisms, posing a risk to agriculture globally, with an estimated annual loss of USD 100–150 billion [3,4,5]. Nematode control, as a result, is a significant obstacle to initiatives to ensure global food security. [6]. Furthermore, PPNs are more difficult to eradicate than are other pathogens because they live in the soil and typically target the subsurface parts of plants [7]. Root-knot nematodes (RKNs, *Meloidogyne* spp.) are widespread and cause significant yield losses in various crops, especially vegetables [8]. *Meloidogyne incognita* is one of the most widely distributed and researched species of *Meloidogyne* [9,10]. *M. incognita* has caused enormous economic losses for Chinese agriculture [11].

Chemical nematicides have been employed to suppress nematodes, but their continued and indiscriminate usage has adverse effects on humans and the environment [7]. Nematicides, such as ethylene dibromide (EDB), methyl bromide, and dibromochloropropane, are no longer widely available in local markets due to the hazards they pose to humans [12]. Nematode control methods are urgently required in light of the rising demand for organic and chemical-free crops [13]. Given the challenges posed by chemical nematicides, the research into innovative, environmentally acceptable alternatives for controlling PPN populations has become more crucial over the last 20 years [6]. The roles of many beneficial soil microorganisms are highly regarded as eco-friendly biological alternatives to synthetic chemical nematicides [14].

Biological control is a nonhazardous method of pest and pathogen management [15,16]. However, the most promising potential chemical nematicide substitutes are antagonists and nematophagous microorganisms [17]. There are few commercially available nematophagous bacteria and fungi for controlling plant-parasitic nematodes [18]. Microorganisms that develop in the rhizosphere protect roots from pathogen assaults and are appropriate for use as biocontrol agents [19]. Nematophagous bacteria are ubiquitous, have extensive host ranges, and are an efficient natural adversary of nematodes [6]. They directly decrease nematode activity by producing antibiotics, toxins, and enzymes; they also compete for resources and space by parasitizing, hence offering a defense response for plant development [20]. Biocontrol agents such as *Clostridium*, *Alcaligenes*, *Serratia*, *Streptomyces*, *Pseudomonas*, *Desulfovibrio*, *Pasteuria*, *Bacillus*, and *Agrobacterium* have demonstrated tremendous promise for biological nematode control [18]. Researchers have previously argued that nonparasitic rhizobacteria are harmful to nematodes, particularly when organic materials have been absorbed into the soil or under anaerobic circumstances [21,22,23]. *Clostridium* species have obvious plant-growth-promoting abilities [24]. Clostridium bacteria release toxins that kill nematodes [25]. Since *Clostridium* has a demonstrated biocontrol impact against a variety of pests and pathogens, it is likely that this species could be employed to control nematode infestations [26,27]. However, no studies have been conducted on using *C. beijerinckii* to suppress nematodes. Thus, to find novel, promising biological control agents against RKNs, we have conducted trials to isolate and screen effective bacterial endophytes against *M. incognita,* and then we selected the effective strain using morphological and molecular approaches. Furthermore, a greenhouse experiment was conducted to assess Sneb518′s potential as a biocontrol agent for *M. incognita* and to investigate the effect of several strains on increasing seed germination and tomato plant growth. This research will provide valuable data on using Sneb518 as a biocontrol agent for RKN management.

## 2. Materials and Methods

### 2.1. Preparation of Bacterial Strain Culture and Nematodes

About 117 rhizosphere soil samples (5 samples were collected from each greenhouse and then combined) were collected randomly from various greenhouses in Liaoning Province to identify and purify a bacterial strain utilizing the soil dilution method. These strains were developed in a beef extract, peptone agar medium (NA) plate, suspended in sterile water, and adjusted to 1.0 × 10^8^ cfu/mL using a hemocytometer under a microscope before being introduced to 50 mL of sterilized lysogeny broth (LB) [28]. Cultures were kept at 28 °C and stirred at 150 rpm for 48 h and were then centrifuged at 6000× *g* for 20 min. The J2s’ lethality and egg hatching bioassays were performed on fermentation filtrate using a 0.22 um filter.

*M. incognita* were maintained in greenhouses at Shenyang Agricultural University on the susceptible tomato cultivar L402. *M. incognita* egg masses were selected under a microscope and surface disinfected for 3 min with 0.5% sodium hypochlorite to decrease microbial contamination, and then rinsed three times with sterile water and incubated at 28 °C to allow the J2s to hatch.

### 2.2. Preliminary Screening on Mortality of Juveniles and Egg Hatchability Treated by Bacterial Strain Filtrate

Approximately 100 newly hatched *M. incognita* J2s were placed in 5 mL glass Petri dishes along with 2 mL of bacterial strain filtrate to determine their impact on J2′s mortality. The control treatment consisted of using sterile water. The tubes were incubated in the dark at 26 °C. The number of hatching J2s was counted using a stereomicroscope. The experiment was repeated three times, with three replicates in each experiment. The following formula was used to calculate the mortality percentage: Mortality (%) = the number of dead J2s/the total number of J2s × 100.

A similar method was used to investigate the influence of bacterial strains on egg hatchability. About 50 surface-sterilized eggs were incubated in 2 mL bacterial strain filtrate for 24 h. A control was performed using 100 surface-sterilized eggs in 2 mL of sterile distilled water. The eggs hatched in the dark at 26 °C. A stereomicroscope was used to count the number of hatching J2s. Each treatment comprised three duplicates, and the experiments were repeated three times. The hatchability of eggs was determined using the following formula:

Egg hatchability (%) = the number of hatched eggs/total number of eggs × 100.

Based on the findings above, the bacterial strain that strongly influenced J2 mortality and egg hatchability was chosen for future investigation.

### 2.3. Second Screening of Sneb518 on Mortality and Egg Hatchability In Vitro

To further investigate the effect of Sneb518 on *M. incognita*, the exposure duration of nematode J2s and eggs in the Sneb518 filtrate was prolonged. As a control, all of the treatments in 2 mL sterile distilled water were utilized. Each treatment had three replicates, and the experiments were repeated three times. The above-mentioned formula was used to calculate mortality and egg hatchability.

### 2.4. Identification of Strain Sneb518

According to Gerhart, et al. [29], physiological and biochemical tests were performed to identify strain Sneb518. Later, Sneb518 was identified further via the phylogenetic analysis of 16S rRNA gene sequences. To extract genomic DNA, standard techniques were used [30,31]. The 27F and 1492R primer pairs were used to amplify the 16S rRNA sequences [32,33]. A PGEM-T vector (Promega, Madison, WI, USA) was modified to include PCR products. The plasmid was extracted and sequenced by the Genewiz Biotechnology Co., Ltd. (Suzhou, China). The 16S rRNA gene sequence was identified using the GenBank database and then aligned with similar species using Clustal X 2.1 [34]. The neighbor-joining strategy was used in Mega 7.0 to create a phylogenetic tree based on the 16S rRNA at 1000 replications [35].

### 2.5. Sneb518′s Effect on Tomato Seedling Growth

Tomato seeds were surface-sterilized with 70% ethanol for 30 s, rinsed three times with sterile distilled water, and air-dried [36]. Seeds were evenly coated with the Sneb518 fermentation at a 70:1 (g/mL) ratio, with distilled water used as a control treatment. The coated seeds were placed in a Petri dish with wet filter paper and cultured for 1 week at 28 ± l °C. The seed germination, shoot, and root length per 10 seeds were measured after 7 days of incubation. The vigor index and germination rate were calculated by Abdul-Baki [37].

Germination rate (%) = number of germinated seeds/the total number of seeds × 100

Vigor index (VI) = (shoot length + root length) × Germination percentage %

### 2.6. Effect of Sneb518 against M. incognita in the Pot Experiments

**Figure 1:**
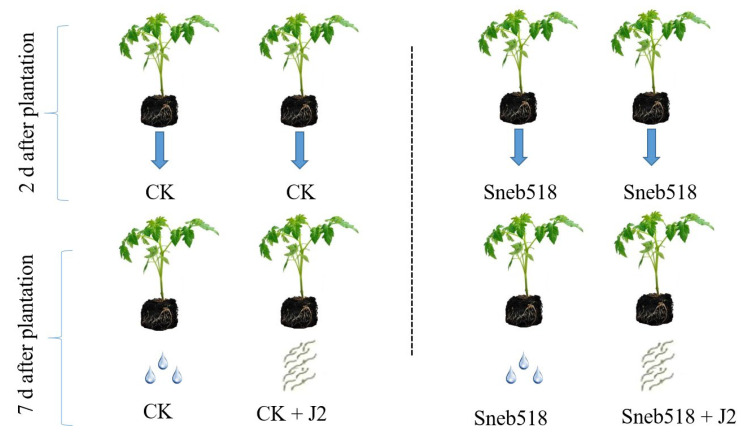
Effect of Sneb518 on the control of *M. incognita* in the pot experiments. A total of four treatments were used. After 2 days of the plantation, the half-plant was inoculated with 20 mL 10× diluted Sneb518 fermentation (Sneb518), while the other half served as a control and was inoculated with sterilized water (CK). After 7 days of the plantation, the 2000 J2s were inoculated into half of the Sneb518 (Sneb518+J2) and half of the control plants (CK +J2).

Tomato seeds were sterilized as above. Sterilized seeds were planted in a seedling tray (5 cm diameter each) and allowed to germinate at 25 °C. Tomato seedlings were transplanted 4 weeks later into pots (12 cm × 12 cm) filled with 500 g sterilized soil and sand (2:1). Plants were placed inside a lighted chamber (16 h photoperiod and temperature range of 23–26 °C). A total of four treatments were used. After 2 days of the plantation, the half-plant was inoculated with 20 mL 10× diluted Sneb518 fermentation (Sneb518), while the other half served as a control and was inoculated with sterilized water (CK). After 7 days of the plantation, the 2000 J2s were inoculated into half of the Sneb518 (Sneb518+J2) and half of the control plants (CK+J2). The nematodes were inoculated through four small, 2 cm deep holes around the roots of the plants. All pots were set in a randomized, complete block form and watered regularly (Figure 1).

Thirty days after inoculation, ten tomato plants were selected randomly for each treatment. The plant roots were rinsed under running water. Plant height, root length, and fresh and dry root weight were measured [38]. The root gall indices were evaluated as described by Barker [39]. The disease index of the root was graded on a scale of 0 to 5, with “0” indicating no gall, “1” suggesting 15% or fewer roots having galls, “2–4” indicating 16–25%, 26–50%, and 51–75% roots having galls, respectively, and “5” indicating >76% roots having galls [39]. The gall indices and biocontrol effectiveness were calculated using the following formulas:

Gall index = ∑No. of diseased plants in each grade × grade/ (total No. plants examined × the highest grade) × 100%

Biocontrol efficacy (%) = (gall index in the control group–gall index in the bacteria treated group)/gall index in the control group × 100.

### 2.7. Statistical Analysis

The statistical analysis was performed using SPSS software (version 20.0). Duncan’s multiple range test (*p* < 0.05) was used to determine the significant difference between treatments.

## 3. Results

### 3.1. Preliminary Screening on Mortality of Juveniles and Egg Hatching Treated by Bacterial Strains In Vitro

A total of 712 bacterial strains were obtained from 117 rhizosphere soil samples via the soil dilution method and screened for potential nematicidal activity against *M. incognita* J2. However, 11 bacterial isolates exhibited intense nematicidal activity (Table 1). At 12 h, the J2 mortality when treated with 11 strains was over 70%, 8 were over 80%, and 1 was over 90%. In terms of egg hatchability, these 11 strains dramatically lowered egg hatchability as well. The hatching rates of Sneb518, Sneb532, Sneb549, Sneb562, Sneb596, Sneb633, and Sneb687 were as low as 10% at 24 h. While Sneb518 showed distinctive inhibition against *M. incognita*, J2 mortality reached 90.73% at 12 h, with eggs hatching at 6.00% at 24 h, and the hatchability for the control was 29.07%. Based on these results, the strain Sneb518 was selected for further study.

### 3.2. Second Screening of Sneb518 for Nematode Mortality and Egg Hatchability

Sneb518 exhibited nematicidal activity against *M. incognita* J2; mortality was 91.55%, 93.99%, and 96.00% after 24 h, 48 h, and 72 h, respectively (Table 2). In contrast, the control group’s mortality rates at 24 h, 48 h, and 72 h were 1.34%, 3.47%, and 5.51%, respectively. *M. incognita* J2s had considerable time-dependent mortality. There was a significant difference between the Sneb518 treatment and the control for all three treatment-time periods.

Hatched juveniles were counted at 48 h, 72 h, and 96 h to determine the impact of Sneb518 on *M. incognita* egg hatching (Table 2). The hatchability of the control was 82.470% at 96 h; however, it was reduced dramatically to 24.12% with the Sneb518 treatment.

### 3.3. Identification of Strain Sneb518

**Figure 2:**
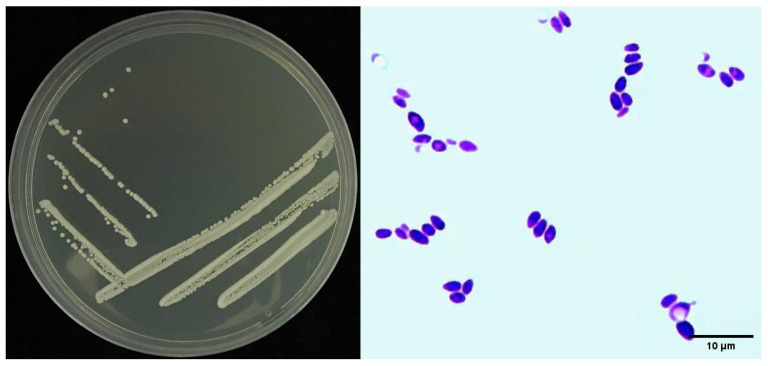
Morphological characteristics of bacteria isolate Sneb518. Left: The morphological characteristics of Sneb518 on NA plate. Right: Gram-positive staining of Sneb518.

The Sneb518 colonies that formed on the NA culture medium were rounded, with irregular edges and bulges in the middle, translucent, grayish white, creamy and sticky, and slightly shiny, with a diameter of about 2 mm at 48 h. The bacterial strain was Gram-positive and had rod-shaped cells (Figure 2).

**Figure 3:**
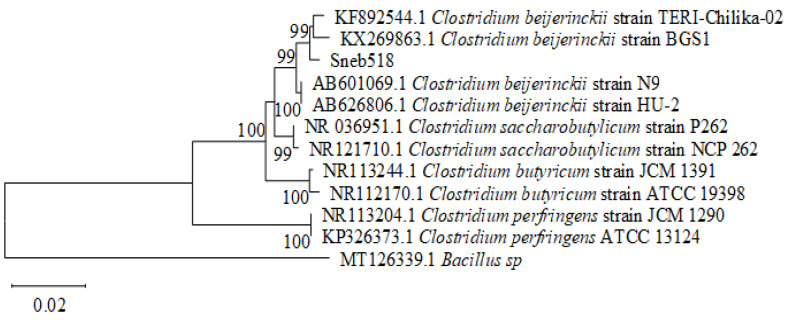
Phylogenetic tree of strain Sneb518 based on the partial nucleotide sequences of 16S rRNA. The tree was constructed using the neighbor-joining method based on a bootstrap analysis of 1000 replicates.

Molecular identification of the bacteria Sneb518 showed that the length of the 16S rRNA sequence, as amplified with primers 27F/1492R, was 1418 bp. In addition, comparing the sequence to BLAST homologous sequences from the GenBank database revealed that the strain Sneb518 had a 98% similarity with *Clostridium beijerinckii.* The phylogenetic tree was constructed with *Clostridium* spp. sequences downloaded from the NCBI database (Figure 3). The strain Sneb518 is clustered on the same branch as *C. beijerinckii* (KF892544 and KX269863). Sneb518 was identified as *C. beijerinckii* based on the findings of morphological and molecular investigations. The sequence of *C. beijerinckii* Sneb518 was submitted to the GenBank database with accession number ON920936.

### 3.4. Sneb518′s Effect on Tomato Seedling Growth

Seed coating was used to evaluate the potential of strain Sneb518 to promote seed germination. Table 3 shows the effect of Sneb518 on tomato seedling growth. A significant difference (*p* < 0.05) was observed in coated seeds with Sneb518 fermentation broth, which increased germination by up to 88.49%. Shoot length and root length demonstrated the potential of Sneb518, which increased by 4% and 20%, respectively, compared to the control. Seed coating with *C beijerinckii* Sneb518 increased the seed vigor index by 60%.

### 3.5. Effect of Sneb518 on the Control of M. incognita in the Pot Experiments

A pot experiment assessed the potential biological control ability of *C. beijerinckii* Sneb518 against *M. incognita*. Thirty days after *M. incognita* inoculation, CK tomato plants exhibited 77.2 root galls, whereas *C. beijerinckii* Sneb518-treated tomato seedlings exhibited 50.26% biocontrol effectiveness as compared to the control (Table 4). Overall, the findings provided strong evidence that *C. beijerinckii* Sneb518 could effectively control *M. incognita* on tomato plants under greenhouse conditions. Moreover, in the present research, *C. beijerinckii* Sneb518 efficiently boosted the growth of plants.

## 4. Discussion

*Clostridium* is the second-largest genus after *Streptomyces*, with over 100 species. It is one of the common bacteria that produce acetate, acetoin, butyrate, hydrogen gas, lactate, carbon dioxide, butanol, acetone, acetyl methyl carbonyl, and ethanol during fermentation [40,41]. Moreover, *Clostridium* species have obvious plant-growth-promoting abilities [24,42]. Toxins produced by *Clostridium* bacteria destroy nematodes [25,43]. *Clostridium beijerinckii* species are abundant in nature and are commonly isolated from soil [44]. Numerous plants and pathogens have reported *Clostridium*’s biocontrol effect against various pests and pathogens, indicating that this genus can be used to control nematode infection [26,27,45]. However, no such research had been performed on the application of *C. beijerinckii* to control nematodes. Keeping that in mind, a novel research study was developed to investigate the nematode-reducing potential of *C. beijerinckii* through seed coating and to increase biomass. Our findings could be a theoretical basis for creating a valuable and marketable biocontrol agent.

Plant-growth-promoting rhizobacteria (PGPR) are a hot issue in the hunt for plant protection against various diseases [46]. Many PGPR strains have been used effectively to treat RKNs [47]. Effective and safe RKN disease control techniques are desperately needed in greenhouses [48,49]. *C. beijerinckii* Sneb518 isolates were selected for further study from 712 bacterial strains found to be effective against RKNs. Sneb518 inhibited juvenile and egg hatching significantly more than other strains. This strain was also tested for its ability to boost plant growth and its biocontrol effectiveness against RKNs through seed treatment in both pot and field experiments. The findings indicated that this Sneb518 strain exhibited biological control ability against *M. incognita*. The biocontrol effect of *C. beijerinckii* Sneb518 might be the consequence of strains promoting plant growth and secreting chemicals that destroy the J2s of nematodes and impede egg hatching (Table 1 and Table 2). Colonization is thought to be a crucial phase in the biological control action of biocontrol bacteria. Secondary metabolites such as enzymes, antibiotics, and poisonous chemicals have been frequently linked to nematicidal activity [50,51]. However, the nematicidal secondary metabolites generated by *C. beijerinckii* Sneb518 have yet to be characterized.

Seed biopriming is the process of coating seeds with microorganisms to protect seedlings against pathogens [52]. Seed treatment was employed as a general approach for applying PGPR that might impart resistance to RKNs in agricultural plant production [53,54]. Seed treatment has been extensively utilized to manage *M. incognita*, *M. javanica*, and *Rotylenchulus reniformis* in various crops, including tomatoes, soybeans, and cotton [55,56,57]. PGPRs showed significant potential as biocontrol agents against *M. incognita* in tomatoes, efficiently promoting plant growth [58]. This study revealed that the application of *C. beijerinckii* Sneb518 as a seed treatment helped to reduce nematode disease and enhance plant growth (Table 3 and Table 4). These data suggested that seed treatment might be an effective and cost-effective economic approach for Sneb518 to control *M. incognita*.

## 5. Conclusions

In conclusion, our findings demonstrated that treating tomato seeds with *C. beijerinckii* Sneb518 considerably decreased the amount of *M. incognita* galls in pot experiments. Furthermore, *C. beijerinckii* Sneb518 killed J2s and reduced egg hatchability in in vitro experiments. Moreover, *C. beijerinckii* Sneb518 also exhibited plant growth characteristics and nematicidal potential in a greenhouse experiment. The findings indicate that *C. beijerinckii* has the potential for microbial application and commercial use as a biocontrol agent in the field. This research will give new options for the sustainable control of root-knot nematode disease in tomatoes and other host plants. Further research is required to assess its biochemical characterization before recommending it as a commercial nematicide. Future research will continue to explore the mechanisms of Sneb518 as a biological control agent for RKNs. This may provide a theoretical basis for the better prevention of RKN disease.

## Figures and Tables

**Table 1:** Preliminary screening results of bacterial strains against *M. incognita*.

Treatments	J2 Mortality (%)	Egg Hatchability (%)
CK	0.99 ± 1.36 ^a^	29.07 ± 3.42 ^f^
Sneb518	90.73 ± 0.74 ^b^	6.00 ± 1.94 ^a^
Sneb528	83.75 ± 5.10 ^c^	11.20 ± 1.19 ^cd^
Sneb532	86.54 ± 6.76 ^d^	6.40 ± 1.21 ^a^
Sneb549	87.87 ± 7.77 ^c^	7.60 ± 1.80 ^ab^
Sneb562	75.91 ± 6.83 ^de^	8.00 ± 1.05 ^ab^
Sneb576	77.29 ± 4.81 ^ef^	13.20 ± 2.76 ^de^
Sneb596	81.30 ± 8.59 ^c^	7.87 ± 1.59 ^ab^
Sneb633	81.60 ± 3.23 ^de^	7.60 ± 2.79 ^ab^
Sneb676	83.67 ± 4.68 ^f^	11.20 ± 2.02 ^cd^
Sneb687	82.68 ± 4.76 ^e^	9.60 ± 1.21 ^bc^
Sneb737	74.37 ± 2.38 ^d^	14.13 ± 1.73 ^e^

Note: Different letters indicates that values are significantly different according to Duncan’s multiple range test at *p* < 0.05.

**Table 2:** Second screening of Sneb518 for J2 mortality and egg hatchability.

Treatments	J2 Mortality (%)			Egg Hatchability (%)		
	24 h	48 h	72 h	48 h	72 h	96
CK	1.34 ± 1.59 ^b^	3.47 ± 3.02 ^b^	5.51 ± 3.11 ^b^	58.93 ± 2.67 ^a^	66.72 ± 3.78 ^a^	82.47 ± 2.71 ^a^
Sneb518	91.55 ± 1.06 ^a^	93.99 ± 0.87 ^a^	96.00 ± 1.16 ^a^	10.27 ± 1.80 ^b^	16.42 ± 3.21 ^b^	24.12 ± 4.34 ^b^

Note: Different letters indicates that values are significantly different according to Duncan’s multiple range test at *p* < 0.05.

**Table 3:** Effect of Sneb518 on the seedling growth of tomato plants.

Treatments	Germination Rate	Shoot Length	Root Length	Seed Vigor Index
CK	82.75 ± 3.89 ^b^	5.73 ± 0.46 ^a^	4.87 ± 0.50 ^a^	8.77 ± 1.03 ^b^
Sneb518	88.49 ± 3.03 ^a^	6.50 ± 0.50 ^a^	5.80 ± 0.26 ^a^	13.90 ± 2.08 ^a^

Note: Different letters indicates that values are significantly different according to Duncan’s multiple range test at *p* < 0.05.

**Table 4:** The biocontrol effect of Sneb518 against *M. incognita* in the pot experiments.

Treatments	Plant Length (cm)	Root Length (cm)	Root Dry Weight (g)	Root Fresh Weight (g)	Gall Index (%)	Biocontrol Efficacy (%)
CK+J2	34.82 ± 3.45 ^b^	15.72 ± 1.14 ^b^	0.31 ± 0.05 ^b^	2.66 ± 0.31 ^b^	77.2 ^b^	-
Sneb518+J2	37.06 ± 3.19 ^a^	16.50 ± 0.75 ^a^	0.34 ± 0.05 ^ab^	2.85 ± 0.09 ^a^	38.4 ^a^	50.26

Note: Different letters indicates that values are significantly different according to Duncan’s multiple range test at *p* < 0.05.

## Data Availability

Not applicable.
